# Ghrelin Ameliorates Angiotensin II-Induced Myocardial Fibrosis by Upregulating Peroxisome Proliferator-Activated Receptor Gamma in Young Male Rats

**DOI:** 10.1155/2018/9897581

**Published:** 2018-08-05

**Authors:** Qian Wang, Xin Sui, Rui Chen, Pei-Yong Ma, Yong-Liang Teng, Tao Ding, Dian-Jun Sui, Ping Yang

**Affiliations:** ^1^Department of Cardiology, China-Japan Union Hospital, Jilin University, Changchun 130033, China; ^2^Jilin Provincial People's Hospital, Changchun 130021, China; ^3^Baohua Hospital of Nongan County, Nongan 130200, China; ^4^College of Pharmacy, Changchun University of Chinese Medicine, Changchun 130021, China; ^5^The First Hospital of Jilin University, Changchun 130021, China; ^6^Traditional Medicine Institute of Jilin Province, Changchun 130021, China

## Abstract

Angiotensin (Ang) II contributes to the formation and development of myocardial fibrosis. Ghrelin, a gut peptide, has demonstrated beneficial effects against cardiovascular disease. In the present study, we explored the effect and related mechanism of Ghrelin on myocardial fibrosis in Ang II-infused rats. Adult Sprague-Dawley (SD) rats were divided into 6 groups: Control, Ang II (200ng/kg/min, microinfusion), Ang II+Ghrelin (100 *μ*g/kg, subcutaneously twice daily), Ang II+Ghrelin+GW9662 (a specific PPAR-*γ* inhibitor, 1 mg/kg/d, orally), Ang II+GW9662, and Ghrelin for 4 wks. In vitro, adult rat cardiac fibroblasts (CFs) were pretreated with or without Ghrelin, Ghrelin+GW9662, or anti-Transforming growth factor (TGF)-*β*1 antibody and then stimulated with or without Ang II (100 nmol/L) for 24 h. Ang II infusion significantly increased myocardial fibrosis, expression of collagen I, collagen III, and TGF-*β*1, as well as TGF-*β*1 downstream proteins p-Smad2, p-Smad3, TRAF6, and p-TAK1 (all p<0.05). Ghrelin attenuated these effects. Similar results were seen in Ang II-stimulated rat cardiac fibroblasts in vitro. In addition, Ghrelin upregulated PPAR-*γ* expression* in vivo* and* in vitro*, and treatment with GW9662 counteracted the effects of Ghrelin. In conclusion, Ghrelin ameliorated Ang II-induced myocardial fibrosis by upregulating PPAR-*γ* and in turn inhibiting TGF-*β*1signaling.

## 1. Introduction

Myocardial fibrosis, a principal pathological basis of ventricular remodeling, is characterized by CFs proliferation and extracellular matrix deposition. Excessive collagen deposition can lead to cardiac dysfunction. Myocardial fibrosis is generally considered a gradually progressive and irreversible process that can finally result in heart failure or fatal arrhythmia [1, 2]. Ang II is a major active component in the renin-angiotensin-aldosterone system and an important stimulating factor of myocardial fibrosis in a variety of cardiovascular diseases. Ang II activation plays an important role in the proliferation, differentiation, and migration of CFs and collagen synthesis by which it contributes to the participation in the formation of myocardial fibrosis [3].

The TGF-*β*1 signaling pathway is activated in myocardium and cardiac fibroblasts of rats induced by Ang II [4]. The TGF-*β*1 signaling pathway includes not only canonical Smad2/3 signaling but also noncanonical signaling. TRAF6 and TAK1 are downstream signals of TGF-*β*1 and belong to noncanonical signaling. Activation of the TGF-*β*1/TRAF6/TAK1 signaling pathway can upregulate connective tissue growth factor (CTGF) expression and contribute to the proliferation of atrial fibroblasts [5].

Ghrelin is the endogenous ligand of the growth hormone secretagogue receptor that has been shown to possess properties that inhibit inflammation, oxidative stress, cell proliferation, and cell apoptosis [6–10]. Ghrelin and its receptor are present in a wide variety of tissues, including the myocardium [11–13]. In heart failure animal models, circulating Ghrelin in the plasma is decreased and there is a negative correlation between plasma Ghrelin levels and the degree of myocardial fibrosis [13, 14]. Li and colleagues demonstrated that Ghrelin and des-octanoyl-Ghrelin treatment markedly protect myocardial injury in rats treated with isoproterenol [15]. Injection of exogenous Ghrelin can also suppress myocardial fibrosis after MI, improving cardiac function [16]. Notably, Ghrelin has been reported to upregulate PPAR-*γ* expression* in vitro *and* in vivo *[17, 18]. Since PPAR-*γ* upregulation can attenuate fibrosis and TGF-*β*1 expression in several other disease models [3, 19], we hypothesize that Ghrelin may ameliorate angiotensin II-induced myocardial fibrosis by activating PPAR-*γ* and inhibiting TGF-*β*1 signaling.

In the present study, SD rat adult rat CFs were used to investigate the role of Ghrelin on myocardial fibrosis induced by Ang II and investigate the molecular mechanisms involved. We demonstrated that Ghrelin plays an antifibrotic role* in vivo* and* in vitro* by upregulating PPAR-*γ* and inhibiting TGF-*β*1 signaling.

## 2. Methods

### 2.1. Animal Experiments

Male SD rats (8 wks old, 210–240 g) were purchased from the Experimental Animal Center of Jilin University. All animal experiments conformed to the Guide for the Care and Use of Laboratory Animals (National Research Council, Eighth edition, 2011) and were approved by the Jilin University Ethics Committee. Rats were subcutaneously injected with Ghrelin (50, 100, or 200 *μ*g/kg, twice daily) with or without Ang II (200 ng/kg/min, microinfusion) for 4 wks. From this pilot examination, Ghrelin at the 100 *μ*g/kg dose effectively reduced protein expression of collagen I and collagen III and increased protein expression of PPAR-*γ*. The 100 *μ*g/kg dose was used for the remainder of experiments.

Rats were divided into six groups (n=6 each): (1) control, (2) Ang II (200 ng/kg/min, microinfusion), (3) Ang II+Ghrelin (100 *μ*g/kg, twice a daily, subcutaneous), (4) Ang II+Ghrelin+GW9662 (1 mg/kg/d, orally), (5) Ang II+GW9662, and (6) Ghrelin. Rats were given Ang II or saline with or without Ghrelin and with or without GW9662 for 4 wks. All rats were fed a standard diet and were provided food and water* ad libitum*.

### 2.2. Echocardiography and Blood Pressure Measurements

Echocardiography was performed 4 wks after Ang II infusion using a Vevo 2100 high-resolution imaging system equipped with a 21-MHz transducer (VisualSonics Toronto, ON, Canada). Following anesthesia with isoflurane, left ventricular internal diameter, and wall thickness (posterior and septal wall), shortening, and ejection fraction were evaluated. Systolic blood pressure was measured every week by tail-cuff method.

### 2.3. Histological Analysis

Rats were euthanized 4 weeks after Ang II infusion, and ventricles sections were stained with Masson's trichrome to assess the degree of cardiac fibrosis. Collagen fraction volume was calculated as the area of green stain divided by the total area of the tissue.

### 2.4. CF Culture

Rat CFs were isolated from left ventricles of adult SD rats (n=5, 8 week old, male). Briefly, the ventricles removed from adult rats were minced into small pieces and digested with collagenase (0.01%). After centrifuging, the single cell suspension was plated for 1.5 h, at which time the media was replaced to remove nonadherent cells. The CFs were incubated in DMEM with 10% fetal bovine serum. When the cells reached 80% confluence, the cells were incubated in serum-free medium for 24 h, after which the cells were stimulated for 24 h.

### 2.5. MTT Assay

Adult rat CFs were cultured in 96-well plates (8,000 cells each well) to 80% confluence, were serum starved for 24 h, and then were pretreated with Ghrelin (1, 10, 100, or 1000 nM) for 24 h before being stimulated with Ang II (100 nM) another 24 h. Similarly, CFs were pretreated with 100 nM Ghrelin for 3, 6, 12, or 24 h before adding Ang II for another 24 h. MTT (5 mg/mL) was added to each well, and the cells were incubated for 6 h at 37°C. The medium was removed, and DMSO was added for 10 min. Absorbance was measured at 490 nm with a microplate spectrophotometer.

### 2.6. Flow Cytometric Analysis

CFs were treated and fixed in 70% ethanol overnight at 4°C. After 24 h of staining with propidium iodide (PI, 50mg/mL in PBS containing 0.1%TritonX-100), approximately 10,000 stained cells were selected and analyzed with ModFit (version2, Verity Software House).

### 2.7. Immunoblotting

Total protein was extracted from ventricular tissue or harvested CFs by homogenizing in a lysis buffer containing 50 mmol/L HEPES, 5 mmol/L EDTA, 100 mmol/L NaCl, 1% Triton X-100, and 1x protease inhibitor cocktail (Roche, Mannheim, Germany) at pH 7.4. Protein concentrations were determined using a modified Bradford assay, and equal amounts of protein were separated by 10% SDS-PAGE gels and transferred onto polyvinylidene difluoride membranes. Membranes were blocked with 5% nonfat milk and incubated with primary antibodies overnight at 4°C. The primary antibodies were collagen I (ab34710, 1:1000, Abcam), collagen III (ab7778, 1:5000, abcam), PPAR*γ* (ab209350, 1:1000, Abcam), p-Smad2 (#18338, 1:1000, Cell Signaling Technology), Smad2 (#5339, 1:1000, Cell Signaling Technology), p-Smad3, 1:1000, Cell Signaling Technology), Smad3 (#9513, 1:1:1000, Cell Signaling Technology), p-TAK1 (#9339, 1:1000, Cell Signaling Technology), TAK1 (#4505, 1:1000, Cell Signaling Technology), TGF-*β*1 (#3711, 1:1000, Cell Signaling Technology), TRAF6 (sc7221, 1:1000, Santa Cruz Biotechnology), and GAPDH (AC001, ABclonal, 1:1000). The membranes were washed and incubated with a secondary antibody (Ab97064, Abcam, 1:5000) for 2 h, and signals were detected using the ECL system (Amersham Life Sciences).

### 2.8. Statistical Analysis

Results are expressed as mean ± SEM. Differences among groups were tested by one-way ANOVA followed by Bonferroni's posttest. A* p value *< 0.05 was considered statistically significant.

## 3. Results

### 3.1. Ghrelin Effects on Collagen I, Collagen III, and PPAR-*γ* Protein Expression in Ang II-Infused Rats

**Figure 1:**
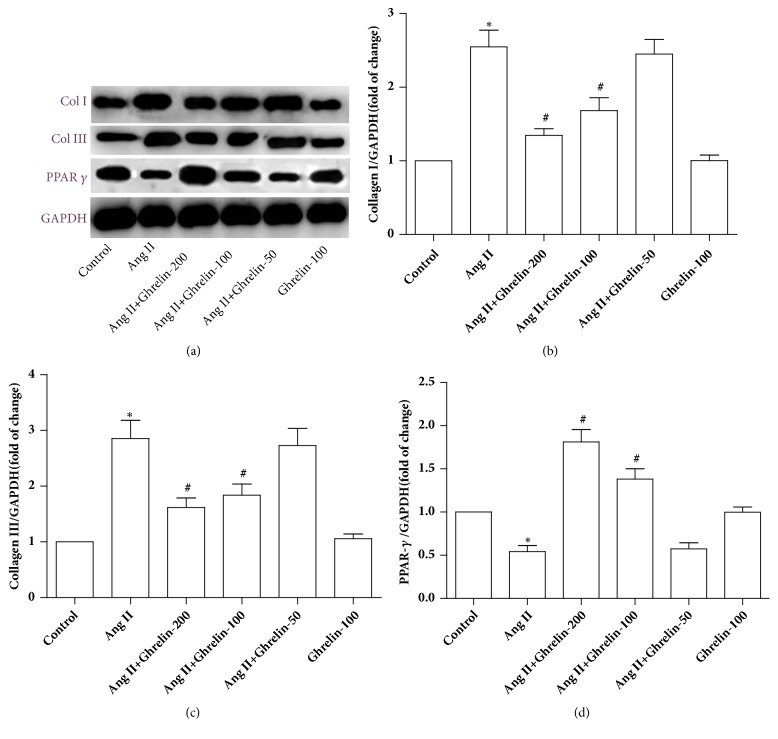
*The effect of different concentrations of Ghrelin on collagen I, collagen III, and PPAR-γ protein expression in Ang II-infused rats*. (a) Collagen I, collagen III, and PPAR**-*γ*** protein expression were assayed by immunoblot. (b, c, and d) Quantitative analysis of (a). Results are expressed as mean ± SEM, n = 6 each per group, ^*∗*^p<0.05 versus Control; ^#^p<0.05 versus Ang II.

Collagen I and collagen III protein expressions were markedly upregulated by Ang II treatment, compared to the untreated control. Ghrelin (100 or 200 *μ*g/kg, but not 50 *μ*g/kg) downregulated collagen I and collagen III protein expression. Ghrelin at these doses also increased PPAR *γ* protein expression. As there was no obvious distinction between the two doses, the 100 *μ*g/kg was used for the remaining experiments. Ghrelin (100 *μ*g/kg) did not affect collagen I, collagen III, and PPAR-*γ* protein expression in saline-infused rats (Figure 1).

### 3.2. Ghrelin Improved AngII-Induced Cardiac Hypertrophy

After 28 days of Ang II infusion, the left ventricular posterior wall thickness in diastole (LVPW; d), left ventricular posterior wall thickness in systole (LVPW; s), intraventricular septal wall thickness in diastole (IVS; d), and intraventricular septal wall thickness in systole (IVS; s) were significantly increased, indicating cardiac hypertrophy. Ghrelin inhibited these changes, and GW9662 counteracted the effect of Ghrelin. Ghrelin had no effect on heart rate (HR), ejection fraction (EF), fractional shortening (FS), or left ventricular internal dimension in diastole (LVID). Ghrelin did not affect wall thickness in saline-infused rats (Table 1).

### 3.3. Ghrelin Improved AngII-Induced Myocardial Fibrosis and Elevations in Blood Pressure

**Figure 2:**
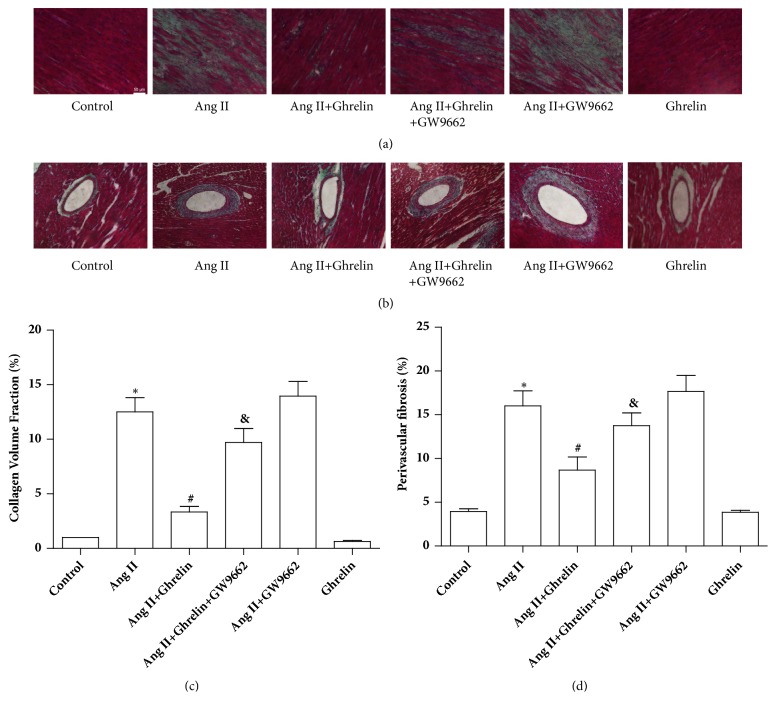
*Ghrelin reduced cardiac fibrosis in Ang II-infused rats*. (a) Masson's trichrome staining to assess interstitial fibrosis in the myocardium. (b) Masson's trichrome staining to assess perivascular fibrosis of myocardium. (c) Quantitation of interstitial fibrosis. (d) Quantitation of perivascular fibrosis. Results are expressed as mean ± SEM, n = 6 each per group, ^*∗*^p<0.05 versus Control; ^#^p<0.05 versus Ang II; ^&^p<0.05 versus Ang II+Ghrelin.

**Figure 3:**
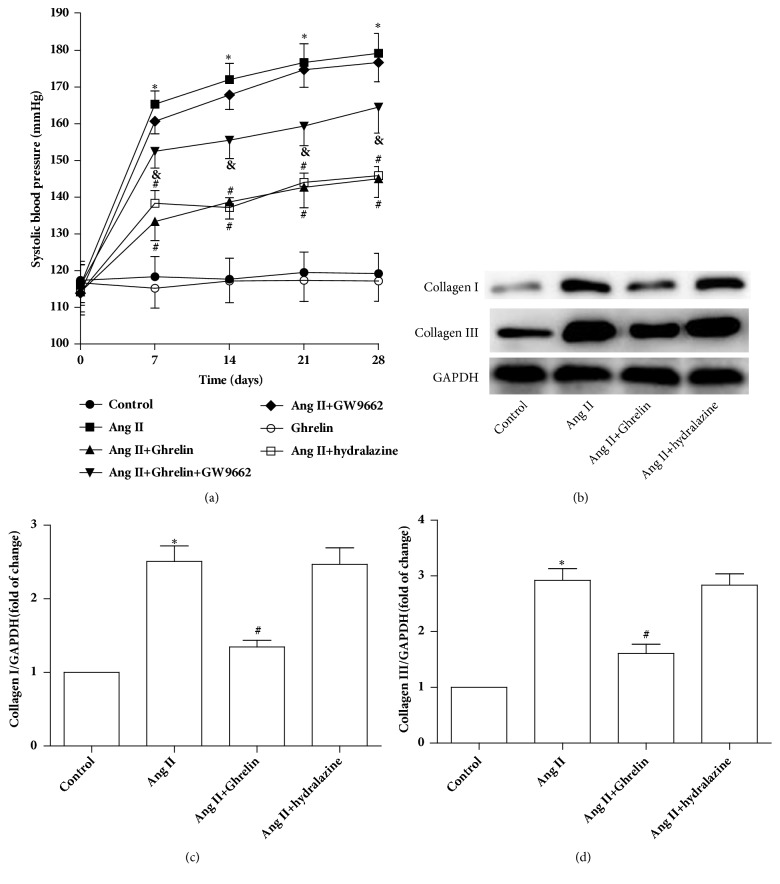
*Ghrelin lowered systolic blood pressure and collagen I and collagen III protein expression in the myocardium of Ang II-infused rats*. (a) Blood pressure tested weekly in control, Ang II, Ang II +Ghrelin, Ang II +Ghrelin +GW9662, Ang II + GW9662, and Ghrelin and Ang II + hydralazine groups by the tail-cuff method. (b) Collagen I and collagen III protein expression in control, Ang II, Ang II +Ghrelin, and Ang II + hydralazine groups. (c and d) Quantitative analysis of (b). Results are expressed as mean ± SEM, n = 6 each per group. ^*∗*^p<0.05 versus Control; #p<0.05 versus Ang II; ^&^p<0.05 versus Ang II+Ghrelin.

Consistent with previous reports [20], the area of cardiac fibrosis in the ventricle was significantly increased following Ang II infusion, compared to control rats. In the Ghrelin plus Ang II treated group, the extent of cardiac fibrosis was attenuated, an effect that was abolished by cotreatment of Ghrelin with GW9662. There was no difference between the degree of fibrosis in the Ang II group and the Ang II+GW9662 group. Injection of Ghrelin alone had no effect on cardiac fibrosis compared to control groups (Figure 2). In addition, systolic blood pressure was significantly elevated, while in rats cotreated with Ghrelin, elevation was partially blocked. To explore whether antifibrosis effects of Ghrelin are related to blood pressure lowering, we administered hydralazine (5mg/kg/d) in drinking water, and it decreased blood pressure at the same level with Ghrelin. There was no statistical difference in collagen I and collagen III protein expression between Ang II groups and Ang II +hydralazine groups (Figure 3). These results indicate the antifibrosis effects of Ghrelin were independent of changes in blood pressure.

### 3.4. Ghrelin Increased PPAR-*γ* Protein Expression and Attenuated TGF-*β*1 and Its Downstream Proteins in Ang II-Infused Rats

**Figure 4:**
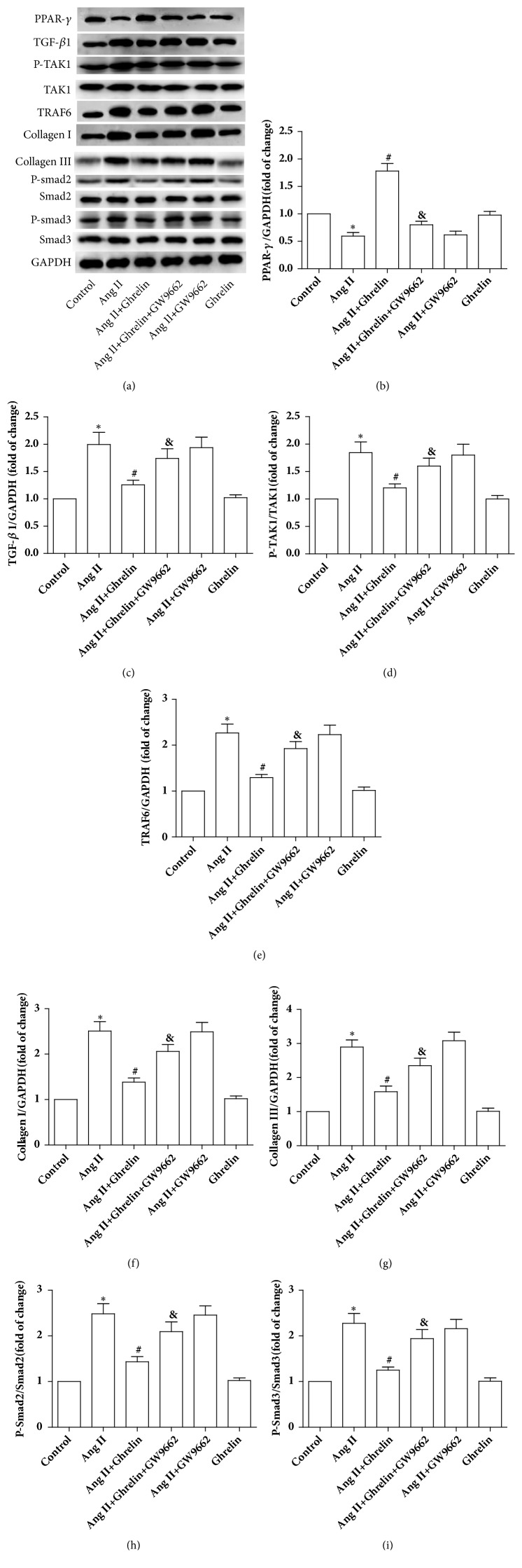
*Ghrelin increased PPAR-γ and attenuated TGF-β1 signaling in Ang II-infused rats*. (a) PPAR-*γ*, TGF-*β*1, P-TAK1, TAK1, TRAF6, collagen I, collagen III, p-Smad2, Smad2, p-Smad3, and Smad3 protein expressions were assayed by immunoblot. (b, c, d, e, f, g, h and i) Quantitative analysis of (a). Results are expressed as mean ± SEM, n = 6 each per group, ^*∗*^p<0.05 versus Control; ^#^p<0.05 versus Ang II; ^&^p<0.05 versus Ang II+Ghrelin.

After Ang II infusion, PPAR-*γ* protein expression significantly decreased compared to untreated rats. Ghrelin treatment markedly elevated PPAR-*γ* levels in the ventricular tissue, while GW9662 abolished this effect. There was no significant difference in PPAR-*γ* expression between Ang II+GW9662 rats and Ang II groups. In addition, the levels of TGF-*β*1, p-Smad2, p-Smad3, p-TAK1, TRAF6, collagen I, and collagen III were significantly higher in the ventricular tissue of the Ang II treated group compared to control rats. Ghrelin treatment reduced the above-mentioned proteins levels, while GW9662 abolished this effect. There was no significant difference in TGF-*β*1 downstream protein expression between Ang II+GW9662 rats and Ang II groups. Ghrelin did not affect protein expression in noninfused rats (Figure 4).

### 3.5. Ghrelin Reduced Ang II-Stimulated CF Viability

**Figure 5:**
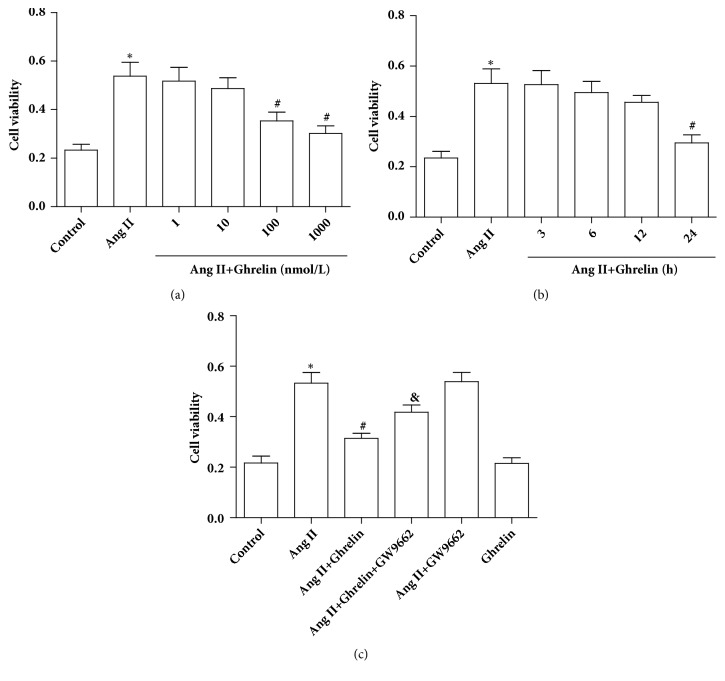
*Ghrelin reduced cell viability of adult rat cardiac fibroblasts stimulated with Ang II*. Cell viability was measured by MTT assay. (a) Cardiac fibroblasts were pretreated with Ghrelin (1, 10, 100, or 1000 nM) for 24 h, followed by stimulation with Ang II (100 nM) for 24 h. (b) Cardiac fibroblasts were pretreated with Ghrelin (100 nM) for 3, 6, 12, or 24 h, followed by stimulation with Ang II (100 nM) for 24 h. (c) CFs were pretreated with or without Ghrelin or Ghrelin plus GW9662 followed by stimulated with or without Ang II for 24h. Results are expressed as mean ± SEM, n = 6 each per group, ^*∗*^p<0.05 versus Control; ^#^p<0.05 versus Ang II; ^&^p<0.05 versus Ang II+Ghrelin.

MTT assay showed that CFs were significantly increased following Ang II stimulation, similar to past reports [21]. Ghrelin given at the 100 nM or 1000 nM doses significantly reduced CF viability induced by Ang II (Figure 5(a)). To assess the optimal timing of Ghrelin treatment, cells were pretreated with ghrelin at 3, 6, 12, and 24 h time points before Ang II stimulation. Only the 24 h pretreatment significantly attenuated CF viability induced by Ang II (Figure 5(b)). In addition, GW9662 treatment interacted with the effect of Ghrelin on CF viability (Figure 5(c)).

### 3.6. Effects of Ghelin on Cell Cycle Distribution

**Figure 6:**
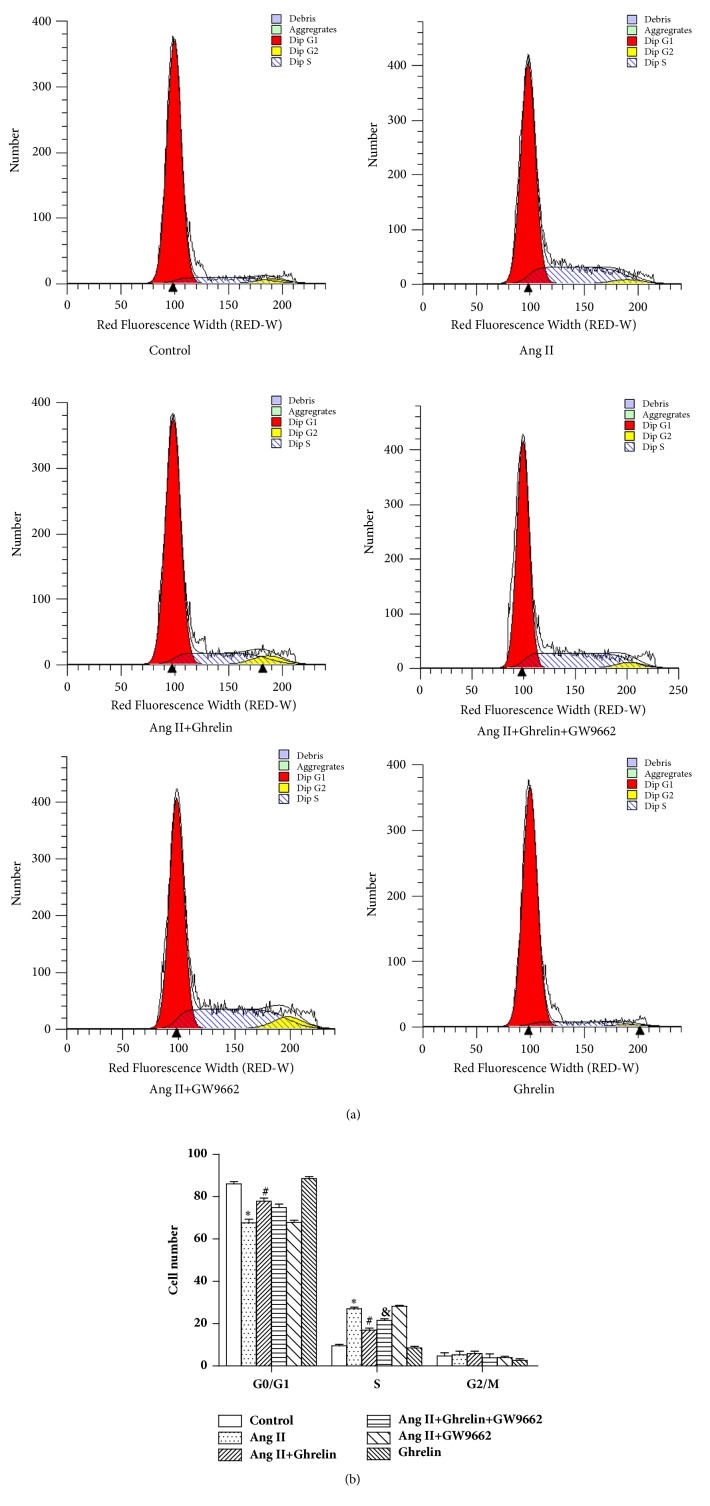
*Ghrelin decreased the number of cardiac fibroblasts stimulated by Ang II that were in the S phase of the cell cycle*. (a) Representative images in the distribution of G0/G1, S, and G2/M phase. (b) Quantitation of the percentages of cell numbers in G0/G1, S, and G2/M phase. Results are expressed as mean ± SEM, n = 6 each per group, ^*∗*^p<0.05 versus Control; ^#^p<0.05 versus Ang II; ^&^p<0.05 versus Ang II+Ghrelin.

**Figure 7:**
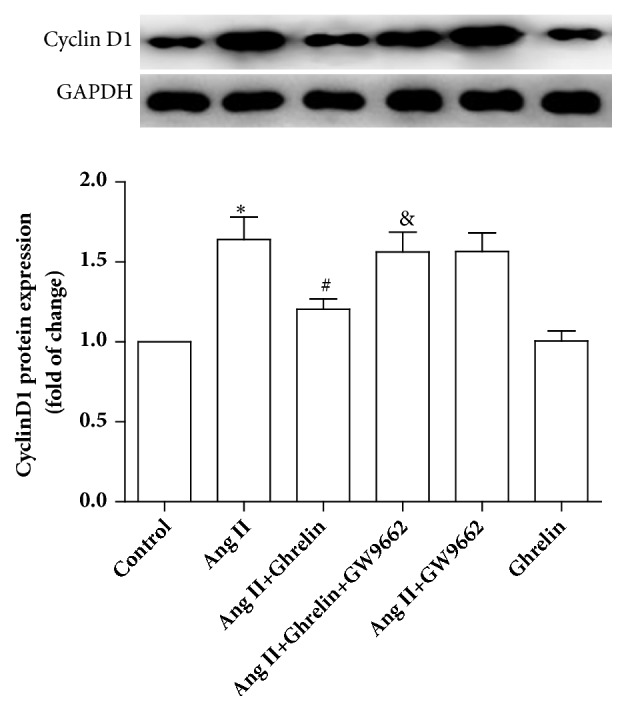
*Ghrelin decreased cyclin D1 protein expression in cardiac fibroblasts stimulated by Ang II*. Cyclin D1 protein expression by immunoblotting in Control, Ang II, Ang II +Ghrelin, Ang II +Ghrelin +GW9662, Ang II +GW9662, and Ghrelin groups. Results are expressed as mean ± SEM, n = 6 each per group, ^*∗*^p<0.05 versus Control; ^#^p<0.05 versus Ang II; ^&^p<0.05 versus Ang II+Ghrelin.

To explore whether the effect of Ghrelin in preventing CFs proliferation was associated with cell cycle regulation, flow cytometry was used to evaluate cell cycle. In the control group, the majority of cells were in G0/G1 phase. In the Ang II treated group, cell numbers in G0/G1 phase decreased, accompanied by increased CFs numbers in S phase. Ghrelin prevented the increase of the number of CFs induced by Ang II in S phase, whereas GW9662 counteracted the effect of Ghrelin. Ghrelin alone had no effect on cell cycle regulation (Figure 6). The cell cycle marker cyclin D1 protein expression showed similar changes (Figure 7).

### 3.7. Ghrelin Increased PPAR-*γ* Protein Expression in Rat CFs Stimulated by Ang II

**Figure 8:**
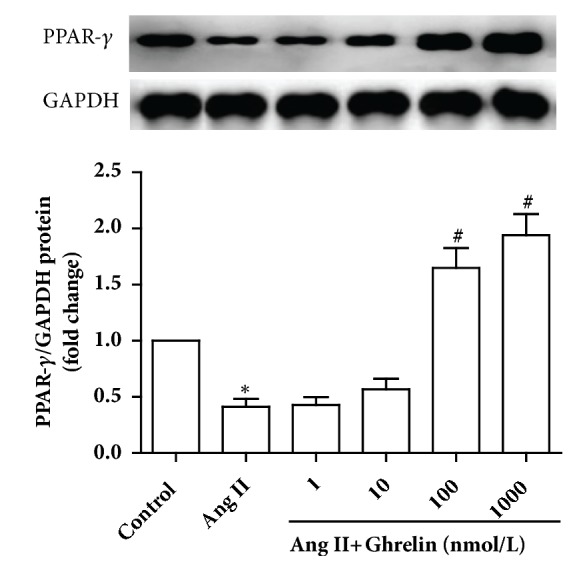
*Ghrelin increased PPAR-γ protein expression in cardiac fibroblasts stimulated by Ang II*. PPAR-*γ* protein expression by immunoblotting in different concentrations of Ghrelin (1, 10, 100, or 1000 nM) pretreatment group. Results are expressed as mean ± SEM, n = 6 each per group, ^*∗*^p<0.05 versus Control; ^#^p<0.05 versus Ang II.

PPAR-*γ* protein expression was significantly lower in CFs stimulated with Ang II, compared to unstimulated controls. This result was in line with past reports [3, 22]. To determine the dose range of Ghrelin effects on PPAR-*γ* expression, CFs were preincubated with Ghrelin at 1,10, 100, or 1000 nM doses for 24 h before stimulation with Ang II for another 24 h. Pretreatment with Ghrelin at 100 or 1000 nM concentrations significantly increased PPAR-*γ* protein expression, which was downregulated by Ang II (Figure 8).

### 3.8. Ghrelin Attenuated TGF-*β*1 Signaling in Rat CFs Stimulated with Ang II

**Figure 9:**
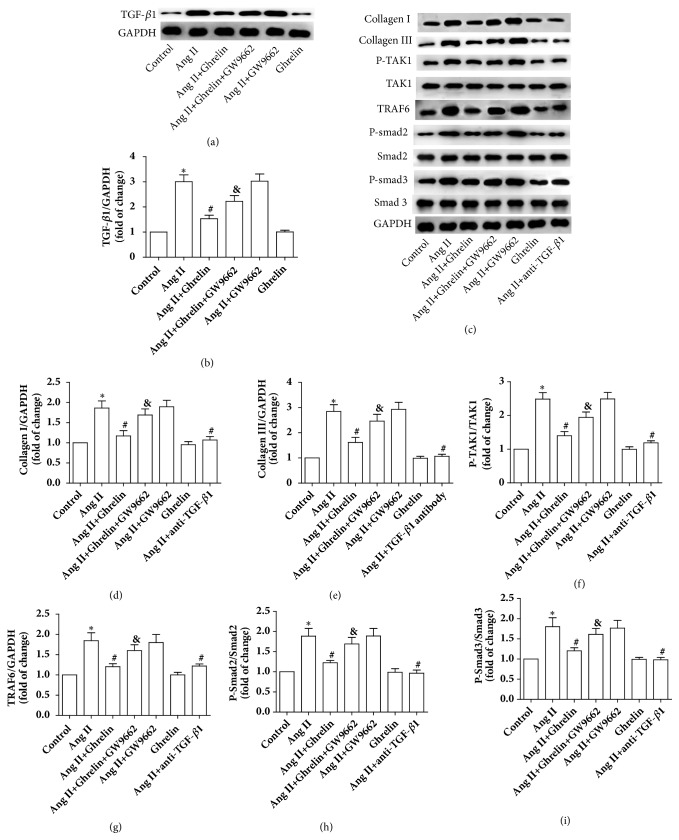
*Ghrelin attenuated TGF-β1 and TGF-β1 signaling in Ang II-stimulated cardiac fibroblasts*. (a) TGF-*β*1 protein was measured in control, Ang II, Ang II+Ghrelin, Ang II+Ghrelin+GW9662, Ang II+GW9662, and Ghrelin groups. (b) Quantitative analysis of (a). (c) Collagen I, collagen III, P-TAK1, TAK1, TRAF6, p-Smad2, Smad2, p-Smad3, and Smad3 protein measured in control, Ang II, Ang II+Ghrelin, Ang II+Ghrelin+GW9662, Ang II+GW9662, Ghrelin, and Ang II+TGF-*β*1 antibody groups. (d, e, f, g, h and i) Quantitative analysis of (c). Results are expressed as mean ± SEM, n = 6 each per group, ^*∗*^p<0.05 versus Control; ^#^p<0.05 versus Ang II; ^&^p<0.05 versus Ang II+Ghrelin.

The protein expression of collagen I, collagen III, TGF-*β*1, p-Smad2, p-Smad3, p-TAK1, and TRAF6 was increased in rat CFs stimulated by Ang II. Ghrelin pretreatment attenuated the level of collagen I, collagen III, TGF-*β*1, p-Smad2, p-Smad3, p-TAK1, and TRAF6 induced by Ang II, while cotreatment of Ghrelin with GW9662 counteracted the effect on collagen I, collagen III, TGF-*β*1, p-Smad2, p-Smad3, p-TAK1, and TRAF6, without affecting total Smad2, Smad3, and TAK1 expression. Interestingly, Ghrelin itself has no effect on the above proteins. In addition, TGF-*β* antibody inhibited the upregulation of collagen I, collagen III, p-Smad2, p-Smad3, p-TAK1, and TRAF6 protein expression induced by Ang II (Figure 9). The results indicate that Ghrelin attenuated collagen secretion and inhibited TGF-*β*1/Smad2/3 and TGF-*β*1/TRAF6/TAK1 signaling by upregulating PPAR-*γ* in rat CFs stimulated by Ang II.

## 4. Discussion

The goal of this study was to determine whether Ghrelin was effective in the inhibition of myocardial fibrosis in the Ang II-infused rats and whether the inhibitory effect of Ghrelin occurred via modulation of the TGF-*β*1 signaling pathways in a PPAR-*γ*-dependent manner. The most significant findings were as follows: (1) Ghrelin treatment decreased myocardial fibrosis and improve myocardial hypertrophy induced by Ang II; (2) Ghrelin treatment increased PPAR-*γ* protein expression and decreased TGF-*β*1 downstream protein expression in Ang II-infused rats;(3) Ghrelin attenuated myocardial fibrosis related protein expression and inhibited CF proliferation in response to Ang II; and (4) GW9662 inhibited these effects of Ghrelin.

Fibrosis occurs in a variety of tissues and organs and is a critical determinant of mortality in these conditions [23, 24]. Myocardial fibrosis is an underlying pathophysiological mechanism in a number of cardiovascular diseases that can accentuate LV remodeling and ultimately progress to heart failure [25]. Therefore, finding effective drugs and exploring mechanisms whereby they prevent, postpone, or regress fibrosis is an important therapeutic goal.

Ghrelin is a gut peptide that has been demonstrated to exert beneficial impact on in a variety of cardiovascular diseases in rodent models [16]. The effect and the underlying mechanisms of Ghrelin in the myocardium of Ang II-infused rats have not been previously reported. In the present experiment, we first demonstrated that Ghrelin inhibited the rise in blood pressure induced by Ang II infusion, and our results were in agreement with the previously reported effect of Ghrelin on human arteries [26]. Ghrelin markedly reduced myocardial collagen deposition following Ang II infusion. However, an equivalent dose of hydralazine that decreased blood pressure to the same degree as Ghrelin had no effect on myocardial fibrosis, indicating Ghrelin exerts antifibrosis effects independent of lowering blood pressure. Ghrelin can upregulate PPAR-*γ* expression in isolated cardiomyocytes stimulated by glucotoxicity and lipotoxicity [17]. PPAR-*γ* is a transcription factor belonging to the nuclear hormone receptor superfamily. Accumulating evidence indicates that PPAR-*γ* exerts a broad range of effects on cardiovascular disease and activation of PPAR-*γ* was beneficial to delay the pathological change of fibrosis [27, 28]. Whether Ghrelin has an antifibrosis effect and whether there is a relationship between Ghrelin and PPAR-*γ* in Ang II-infused rats and Ang II-stimulated CFs had not been previously evaluated. In the present study, Ghrelin induced expression of PPAR-*γ*, and GW9662 counteracted the effect of Ghrelin on PPAR-*γ* expression and myocardial fibrosis stimulated by Ang II, indicating Ghrelin decreases myocardial fibrosis through PPAR-*γ*.

Maejima and colleagues demonstrated that telmisartan, a partial PPAR-*γ* agonist, could reduce TGF-*β*1 expression post-MI by activating PPAR-*γ* in the noninfarcted myocardium in rats [28]. TGF-*β*1 plays an important role in the development cardiac fibrosis [29]. The canonical TGF-*β*1 and Smad2/3 signaling pathway can promote ECM production in Ang II-stimulated CFs; however, the PPAR-*γ* agonist curcumin could suppress ECM production by activating PPAR-*γ* [19]. The noncanonical TGF-*β*1 signaling pathway includes TRAF6 [30]. TRAF6 is prominently expressed in myocardial tissue [31], and activation of TRAF6 was shown to contribute to TAK1 ubiquitination, followed by an accelerated development of cardiac hypertrophy and fibrosis in a mouse aortic banding model [32]. TAK1 is a mitogen-activated protein kinase kinase kinase (MAPKKK) downstream of TRAF6 [32, 33]. Therapies that limit fibrosis have been shown to decrease TAK1 expression, consistent with its role in signaling [29]. Since Ghrelin upregulated PPAR-*γ* expression, we further explored the* in vitro* and* in vivo* linkage between Ghrelin and TGF-*β*1 signaling. Our results indicated that Ghrelin inhibited TGF-*β*1 as well as proteins downstream of TGF-*β*1 in a PPAR-*γ*-dependent manner. TGF-*β*1 inhibition decreased p-smad2, p-smad3, p-TAK1, and TRAF6 protein expression. The effect of the anti-TGF-b1 antibody was similar to Ghrelin.

Ghrelin can inhibit smooth muscle cell proliferation stimulated by angiotensin II by preventing cAMP/PKA signaling [34]. CFs are the most numerous cell type in the rat myocardium, and CFs are the major producers of extracellular matrix [35–37]. Ang II is a strong profibrogenic factor [21, 38]. Limiting Ang II-induced CFs proliferation can inhibit the formation and development of myocardial fibrosis [39]. The current experiment data indicate that Ghrelin prevents CFs proliferation and blocking the entrance of cells from G0/G1 to S phase by PPAR-*γ* activation in vitro.

In this study, young male rats were used. Therefore, the results cannot be extrapolated to older or female cohorts. Whether the effects of Ghrelin on myocardial fibrosis induced by Ang II changes with age or sex needs to be explored.

## 5. Conclusions

In summary, Ghrelin inhibited Ang II-induced cardiac fibrosis in a PPAR*γ*-dependent manner in vivo and in vitro. Because RAS system activation and myocardial fibrosis play an important role in the formation and development of heart failure, evaluating the effects of novel interventions such as Ghrelin is warranted.

## Figures and Tables

**Table 1:** Echocardiography results.

	Control	Ang II	Ang II+ Ghrelin	AngII+Ghrelin +GW9662	AngII+ GW9662	Ghrelin
Echocardiography						
HR (bpm)	389 ± 9	421 ± 11	404 ± 6	411 ± 11	422 ± 10	383 ± 7
EF (%)	77 ± 1.73	85 ± 1.50	81 ± 2.08	84 ± 1.35	85 ± 1.41	78 ± 1.74
FS (%)	38 ± 1.84	42 ± 2.21	40 ± 1.86	42 ± 2.37	42 ± 1.89	39 ± 1.96
LVPW; d (mm)	1.81 ± 0.07	2.38 ± 0.08^*^	1.9 ± 0.09^#^	2.30 ± 0.11^&^	2.38 ± 0.10	1.85 ± 0.07
LVPW; s (mm)	3.36 ± 0.11	4.05 ± 0.15^*^	3.49 ± 0.10^#^	3.98 ± 0.14^&^	4.06 ± 0.15	3.43 ± 0.09
LVID; d (mm)	6.74 ± 0.21	5.82 ± 0.23	6.31 ± 0.20	5.84 ± 0.22	5.65 ± 0.21	6.85 ± 0.22
LVID; s (mm)	3.28 ± 0.17	2.50 ± 0.20	2.83 ± 0.23	2.58 ±0.21	2.41 ± 0.18	3.29 ± 0.16
IVS; d (mm)	1.71 ± 0.07	2.25 ± 0.09^*^	1.81 ± 0.05^#^	2.12 ±0.06^&^	2.23 ± 0.11	1.73 ±0.06
IVS; s (mm)	3.32 ± 0.10	3.88 ± 0.14^*^	3.45 ± 0.08^#^	3.82 ± 0.01^&^	3.92 ± 0.11	3.36 ± 0.07

Ang II, angiotensin II; HR, heart rate; EF, ejection fraction; FS, fraction shortening; LVPW; d,l eft ventricular posterior wall thickness in diastole; LVPW; s, left ventricular posterior wall thickness in systole; LVID; d, left ventricular internal dimension in diastole; LVID; s, left ventricular internal dimension in systole; IVS; d, intraventricular septal thickness in diastole; IVS; s, intraventricular septal thickness in systole. Data are means ± SEM. ^*^P < 0.05 versus Control.^ #^P < 0.05 versus Ang II; ^&^P < 0.05 versus Ang II+Ghrelin.

## Data Availability

The data used to support the findings of this study are included within the article.
